# Mechanical and Thermal Stress Analysis of Cervical Resin Composite Restorations Containing Different Ratios of Zinc Oxide Nanoparticles: A 3D Finite Element Study

**DOI:** 10.3390/ma15165504

**Published:** 2022-08-10

**Authors:** Negar Yazdani, Hossein Ashrafi, Mutlu Özcan, Negin Nekoueimehr, Mohsen Kholdi, Azin Farzad

**Affiliations:** 1 Faculty of Dentistry, Kashan University of Medical Sciences, Kashan 8715988141, Iran; 2 Department of Applied Design, Faculty of Mechanical Engineering, University of Kashan, Kashan 8731753153, Iran; 3 Division of Dental Biomaterials, Clinic for Reconstructive Dentistry, University of Zürich, CH-8032 Zürich, Switzerland; 4 Department of Solid Mechanic, Faculty of Mechanical Engineering, University of Kashan, Kashan 8731753153, Iran

**Keywords:** cervical lesions, finite element method, composite restorations, zinc oxide nanoparticles, thermal stress distribution, mechanical stress distribution, dental materials

## Abstract

Due to an increase in prevalence of cervical lesions, it is important to use appropriate restorative materials to reduce the incidence of secondary lesions. Owing to having antibacterial properties, cervical composite restorations containing different ratios of Zinc Oxide nanoparticles (ZnO NPs) have been analyzed using the Finite Element method to determine the optimal incorporation ratio from mechanical and thermal perspectives. A numerical simulation is conducted for a mandibular first premolar with a cervical lesion (1.5 × 2 × 3 mm^3^) restored with composites containing 0 to 5% wt. ZnO NPs. Subsequently, the samples are exposed to different thermo-mechanical boundary conditions, and stress distributions at different margins are examined. The accumulated stress in the restoration part increases for the 1% wt. sample, whereas the higher percentage of ZnO NPs leads to the reduction of stress values. In terms of different loading conditions, the least and most stress values in the restoration part are observed in central loading and lingually oblique force, respectively. The change in the surface temperature is inversely correlated with the ratio of ZnO NPs. In conclusion, the composite containing 5% wt. ZnO NPs showed the most proper thermo-mechanical behavior among all samples.

## 1. Introduction

In the last decades, life expectancy has greatly improved. That results in a significant increase in multiple chronic diseases, polypharmacy, their interactions, and their adverse effects. Xerostomia and differing normal bacterial flora of oral cavities are some of those effects which contribute to masticatory and nutritional problems due to an increase in dental caries and periodontal disease [1,2,3]. In addition, non-carious cervical lesions (NCCLs) have high prevalence (63%) in these communities. A positive correlation is revealed between the increased value of NCCLs and aging [4]. NCCLs may occur in any tooth. However, there is a high prevalence of NCCLs in first premolars [5]. It is believed that this is primarily due to the higher prevalence of early contact and limited protection by saliva in premolars [6]. In the case of treating these lesions with inappropriate restorative materials and techniques, secondary lesions are predictable. Debonding, leakage, and retention failure of the restoration mostly result from biofilm accumulation and improper stress distribution [7,8]. The previous studies show that the employment of appropriate restorative materials is able to mitigate biofilm accumulation, reduce the risk of periodontal diseases and occurrence of caries, as well as cause less stress distribution on pulp tissue [9]. Otherwise, it may increase the likelihood of secondary lesions, failure in restorations, and the cost of treatment imposed on the patient [10].

A wide range of studies are conducted to introduce the most proper restorative material for cervical restorations. Some research studies recommend amalgam [11] and microfilled composites [12] as the choice material. However, several studies acknowledge resin-modified glass ionomer as the best material for restoring cervical lesions [10,13,14,15]. On the other hand, there are also some studies that indicate no statistically significant difference between NCCLs restored with RMGI, packable and flowable composites. They considered all investigated materials as acceptable treatments for NCCL in terms of fracture resistance and microleakage [16]. Clinical investigations demonstrate that the type of composite material seems to have no significant effect on the clinical performance of cervical restorations, while higher retention rates are obtained in RMGI compared with composite resin [17,18]. 

Various nanoparticles are added to dental materials to increase their pros, which have different effects on their “thermo-mechanical” properties [19,20]. Zinc oxide nanoparticles (ZnO NPs) are metal oxide nanoparticles that are widely and diversely used in dentistry in recent decades. Its wide application is due to demonstrating some eye-catching properties such as having antimicrobial effects, reducing microleakage of resin-based treatments, improving the hardness of dental materials, showing anti-sensitivity effects, promoting angiogenesis and dentine remineralization, preventing biofilm development, etc. [21]. Hence, proper antimicrobial, chemical, physical, mechanical, and optical properties of ZnO NPs open new opportunities when applied in dentistry [22,23,24]. Modifying the components of dental materials could affect various properties of the final product. Thus, new properties of materials containing ZnO NPs should be precisely evaluated before clinical application. Prior investigations suggest that incorporating ZnO NPs with dental materials results in an increase [22,25], decrease [26], or no significant difference [27,28] in their mechanical properties.

The variation in the properties of materials has potential to affect the stress distribution in the tooth and, thereby, the durability of the restoration. The literature shows that a lower stress concentration in the tooth-restoration complex is a good indicator for an appropriate material [11,29,30]. To date, various methods have been developed and employed to measure distributed stress in tooth structure. The finite element method (FEM) is acknowledged as a reliable numerical tool that is widely used in the literature for simulation and computational analysis [31]. FEM is used in dentistry to observe stress distributions theoretically within structures, which is a challenge in other conventional methods [32]. FEM provides researchers with the capability of static and dynamic analyses in a non-invasive way under various corresponding variables [33,34]. Yet, FEM has some limitations: (1) difficulties in simulating biological dynamics, (2) higher risk of human-based errors, and (3) extensive computation time due to the complexity of numerical calculations [11,31].

The aim of this study is to examine the stress distribution in mandibular first premolar with cervical composite restorations containing different ratios of ZnO NPs under various thermo-mechanical boundary conditions. To this end, the study is investigated under various occlusal forces and thermal situations using 3D-FEM. Finally, the optimal incorporation ratio is assessed considering the maximum effective values of von Mises stress (VMS) and maximum principal stress (MPS) accumulated in various points on the tooth-restoration complex.

## 2. Materials and Methods

The tooth that is investigated in this study is selected based on having higher prevalence of cervical lesions. Therefore, an intact mandibular first premolar with fully formed root and without any cracks, fractures, or caries is selected. A CT scan image is acquired with 0.5 mm interval cross sections using a spiral computed tomography scanner (Aquillion 16, Toshiba, Tokyo, Japan). These cross sections are acquired in digital imaging and communication in medicine format. In each cross section, different layers including enamel, dentin, and pulp are accurately determined. This information is transferred to Solidworks software 2020 and converted into a 3D model.

Different boundaries of the tooth are determined. The alveolar bone is designed 3 mm below the cementoenamel junction, mimicking the morphology of bone in the premolar region. The cancellous bone and its stress absorber impact are neglected in order to obtain more obvious stress distribution patterns in the tooth-restoration complex and critical regions with higher accumulated stress (as an indicator for possibility of failure). This was accepted due to the fact that omitting the deformability of the bone results in higher stress concentrations in the root portion of the tooth and, thereby, in the tooth-restoration complex [30]. A 0.3 mm shell between the root and the surrounding bone is simulated as the periodontal ligament. It is previously demonstrated that considering the pulp in the FE model has no effect on the nodal cervical stress values [35]. So, the pulp and cementum are not modeled owing to negligibly low Young’s modulus and similar structure to dentin, respectively.

**Figure 1:**
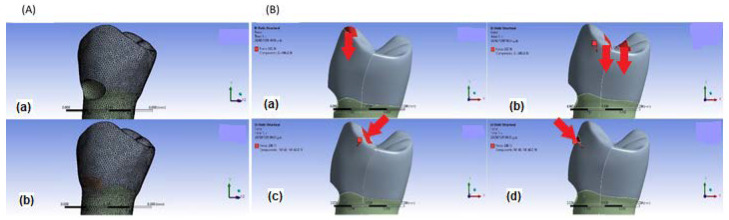
(**A**) Finite element meshed models of mandibular first premolar tooth. (**a**) Unrestored model, (**b**) Restored model. (**B**) The finite element model under the various loading sites; the direction and location of occlusal forces are illustrated with red regions and arrows on the tooth surface. (**a**) Buc.T, (**b**) Cent, (**c**) Buc.L, (**d**) Buc.B.

The model file is transferred as a STEP file to ANSYS Workbench 2020 R1. A piece of tooth in the cervical region with 1.5 × 2 × 3 mm^3^ (1 mm in the enamel) is separated by the slice tool in the static structural environment. The internal line angles of the remaining cavity are rounded. Then, the 3D FE model (see Figure 1A) is created by discretization in the model environment. It should be noted that this model contains 434,196 tetrahedron elements and 688,549 nodes. The meshes have an average dimension of 0.2 mm. The average value for the aspect ratio is 1.9515 (standard deviation = 1.2952). All materials are considered isotropic, homogeneous, and have linear elastic properties. The mechanical and thermal properties that are used in this study (reported in Table 1) come from the previous works [25,36,37,38,39]. Due to the lack of comprehensive available data, laboratory verification of conductivity of the intended materials is supplemented. A mesh convergence test with an error of less than 10% is performed to ensure that the mesh does not affect the results.

The restorative materials that are investigated in this study are resin composite Heliomolar Flow (Ivoclar Vivadent AG/FL-9494 Schaan/Liechtenstein) containing 0, 1, 2, 3, and 5 wt.% ZnO NPs. To simulate mechanical boundary conditions, a fixed support is considered at the root section that is assumably located in bone. Then, four loading conditions are applied at 36.5 °C based on normal occlusal and hyper-functional forces in the oral cavity; see Figure 1B [35,40,41,42,43]: (1) Cent: two parallel 100 N forces applied at a distance of 1 mm from the central groove, (2) Buc.L: a 200 N lingually oriented force (45°) applied at 1 mm below the buccal cusp tip, (3) Buc.B: a 200 N buccally oriented force (45°) applied at 1 mm below the buccal cusp tip, and (4) Buc.T: a 200 N force applied at the buccal cusp tip.

In addition, a steady-state thermal FEA is performed in order to investigate thermal behavior of the tooth-restoration complex. Boundary conditions are simulated by contacting two 60 °C and 4 °C liquid flows with the outer surface of tooth, while the temperature of inner structures is maintained at 36.5 °C. The chosen temperatures are obtained within the reported range of the intraorally produced temperature extremes [44].

Considering these mechanical and thermal boundary conditions, the VMS and MPS values (in MPa) are investigated at six probe sites: 1- occlusal (upper) margin, 2- gingival (lower) margin, 3- distal (left) margin, 4- mesial (right) margin, 5- depth of the cavity at tooth-restoration interface, and 6- the restoration part. Finally, the accumulated stress values are analyzed by ANSYS Workbench 2020 R1 software and potential failure areas are assessed.

## 3. Results

This section outlines the results regarding the von Mises stress analysis (see Section 3.1) and maximum principal stress (see Section 3.2). In each section, the mechanical and thermal stress analyses are presented.

### 3.1. Von Mises Stress Analysis

The VMS value is a good indicator for measuring the possibility of failure [18]. The results regarding the accumulated stress at various probe sites are observed and reported in the following of this section.

#### 3.1.1. Mechanical Stress Analysis

**Figure 2:**
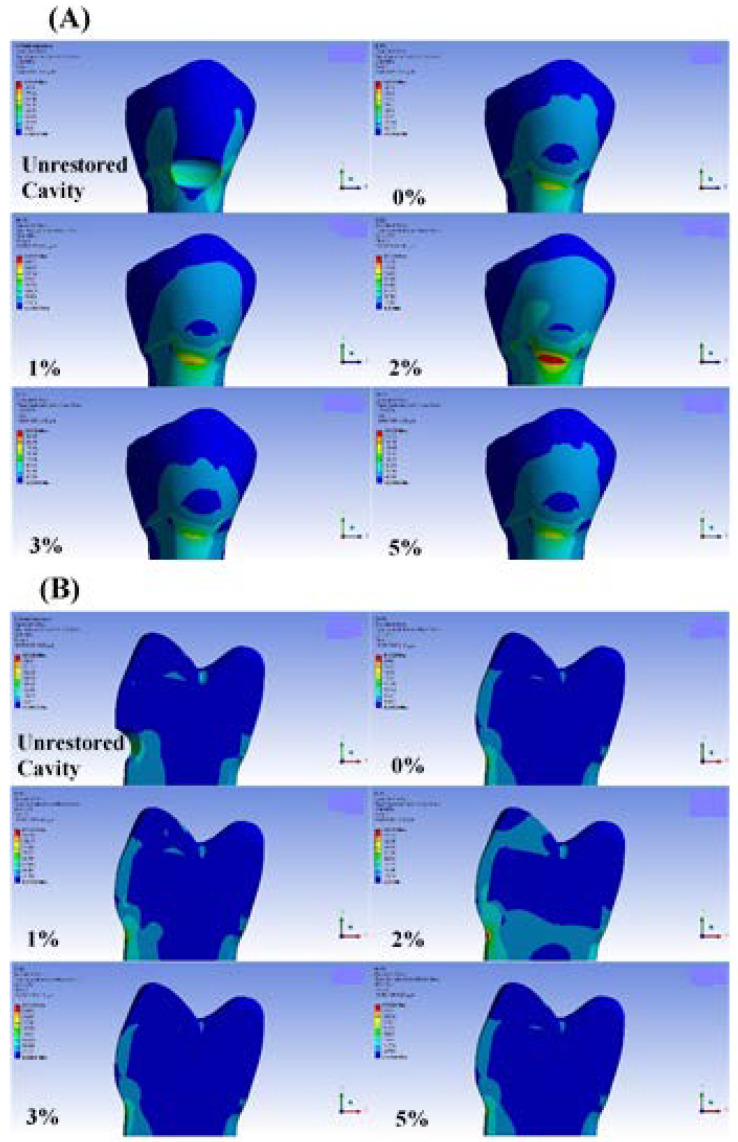
Von Mises stress distribution (MPa) in Buc.L loading site. (**A**) Buccal view, (**B**) Mid-cut view. similar patterns of stress distribution with different magnitudes are observed among various loading conditions.

Figure 2 shows VMS distribution in the Buc.L loading condition. A similar pattern of stress distribution is observed in the tooth-restoration complex at the cervical region under different loading sites (the actual pattern of stress distribution in various boundary conditions can be found in Supplementary Materials, Figures S1–S5). In contrast, the magnitude of stress values differs among various boundary conditions. The highest stress values in restoration part are obtained in the Buc.L group (Buc.L > Buc.T > Buc.B > Cent), while the highest stress values in cavity margins are identified in the Buc.B loading condition.

Considering the stress distribution in unrestored lesions, stresses are mostly concentrated at lateral margins and cavity depth, regardless of loading condition. It is evident that after restoring the cavity, stresses accumulated in lateral margins and cavity depth are decreased. However, it is observed that VMS values are increased at gingival and occlusal margins. The increase in stress value at the gingival margin is more dramatic compared to other margins.

**Figure 3:**
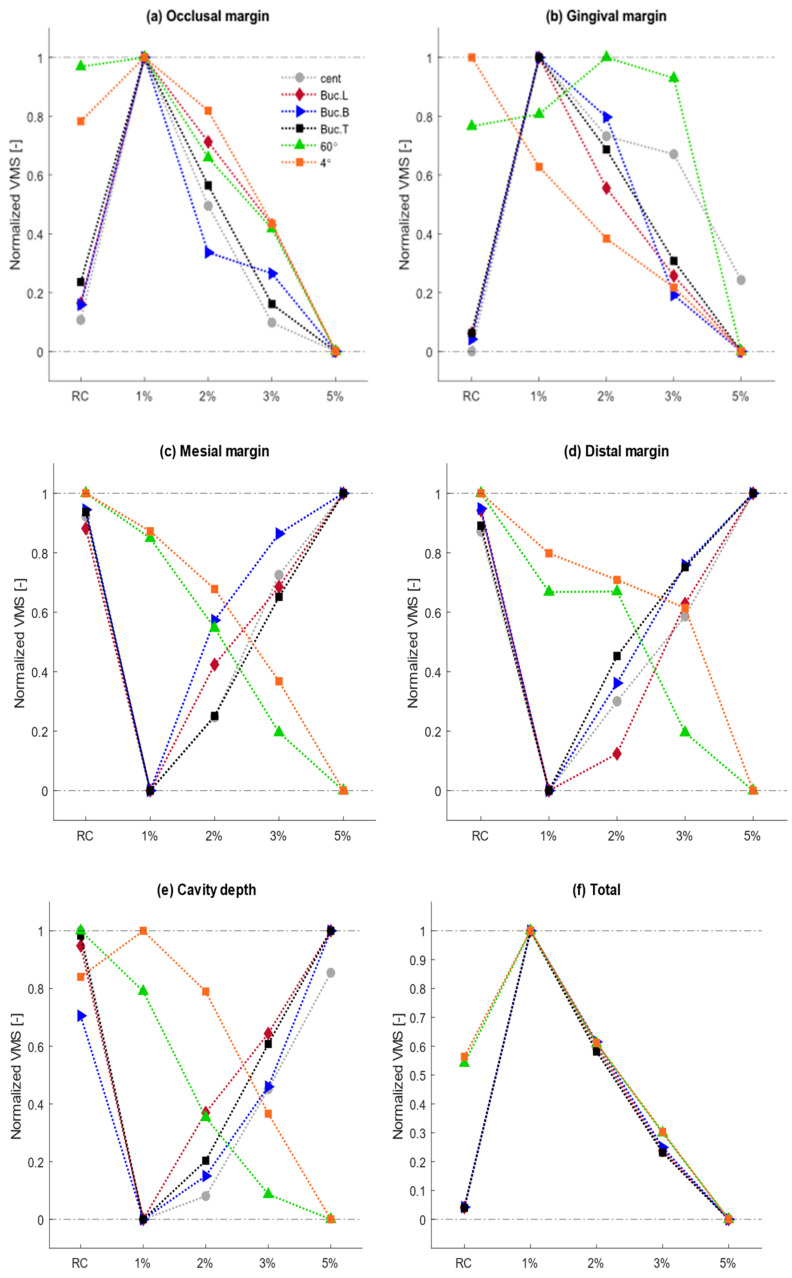
Normalized changing trend of VMS values created among various incorporation ratios of ZnO NPs in various boundary conditions.

Figure 3 shows that as the ZnO content is increased in the resin composite, it results in identical patterns for stress values at various probe sites. The changing trend in stress values is somehow similar among various loading sites. However, the magnitude of VMS values demonstrates huge differences in various boundary conditions. For instance, the Buc.L force creates maximum VMS values in the restoration part (~200 MPa), whereas the maximum stress due to the Cent force is about 18 MPa. Therefore, the impact of increasing the ZnO ratio on stress changing trends could not be clearly observed using absolute values for the corresponding loading conditions. This is why the absolute stress values are normalized with respect to the lowest and highest values for corresponding boundary conditions. Normalization allows us to conduct a better analysis to find the correlation between VMS values and ZnO content. The normalized changing trends occurring at six specified probe sites are demonstrated in Figure 3. Normalized stress values properly illustrate the difference in accumulated stress among studied samples, where zero and one in the vertical axis demonstrates the minimum and maximum stress values observed among studied groups in each loading site, respectively. Further, the absolute values under various boundary conditions are also reported (see Table S1).

#### 3.1.2. Thermal Stress Analysis

It is worth mentioning that in both thermal conditions, the VMS values in the unrestored cavity are significantly lower than these stresses in the restored models, regardless of material type. Although the stresses in each margin are not significantly different among various materials, a general trend is observed by increasing the incorporation ratio after normalizing the VMS values (see Figure 3).

Furthermore, our results indicate that, in the unrestored cavity, hot stimuli create higher VMS values compared with cold ones. However, in restored models, higher accumulated stresses are observed in cold exposure rather than hot exposure.

#### 3.1.3. Temperature Assessment

The range of surface temperature changes shows an inverse relation with ZnO NP content. In hot exposure, the maximum and the minimum surface temperatures are observed in the control (53.587 °C) and the 5 wt.% sample (51.523 °C), respectively. On the other hand, in the cold stimuli, the control (13.596 °C) and the 5 wt.% model (16.363 °C) show, in sequence, the minimum and maximum surface temperatures. Unlike the surface, the temperature in the cavity depth remained constant in all cases (36.5 °C).

### 3.2. Maximum Principal Stress Analysis

Considering the brittle structure of the tooth, MPS is reported as a proper indicator for assessing stress values at a particular area. Thus, the distributed stresses at studied probe sites are reported in MPS values as well. The observed data based on MPS values are reported in the following of this section.

#### 3.2.1. Mechanical Stress Analysis

**Figure 4:**
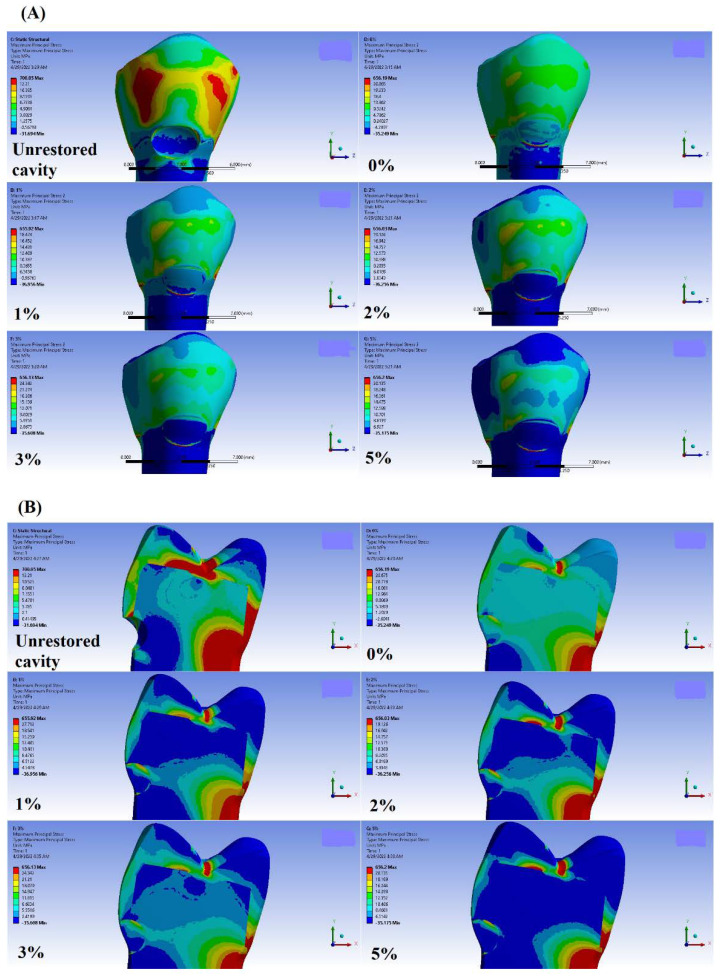
Maximum Principal Stress distribution (MPa) in Buc.L loading site. (**A**) Buccal view, (**B**) Mid-cut view. similar patterns of stress distribution with different magnitudes are observed among various loading conditions.

A similar pattern of MPS distribution in the tooth-restoration complex is observed under different loading conditions. Due to its higher stress values at the restoration part, the Buc.L loading condition is selected to demonstrate this pattern of MPS distribution (see Figure 4). In the unrestored cavity, the highest stress is accumulated at the occlusal margin, regardless of loading condition. A notable finding is that in unrestored lesions, the stresses accumulated at cavity depth are compressive due to having negative MPS values, whereas the tensile stress concentration is observed in other margins of the cavity (positive MPS values).

**Figure 5:**
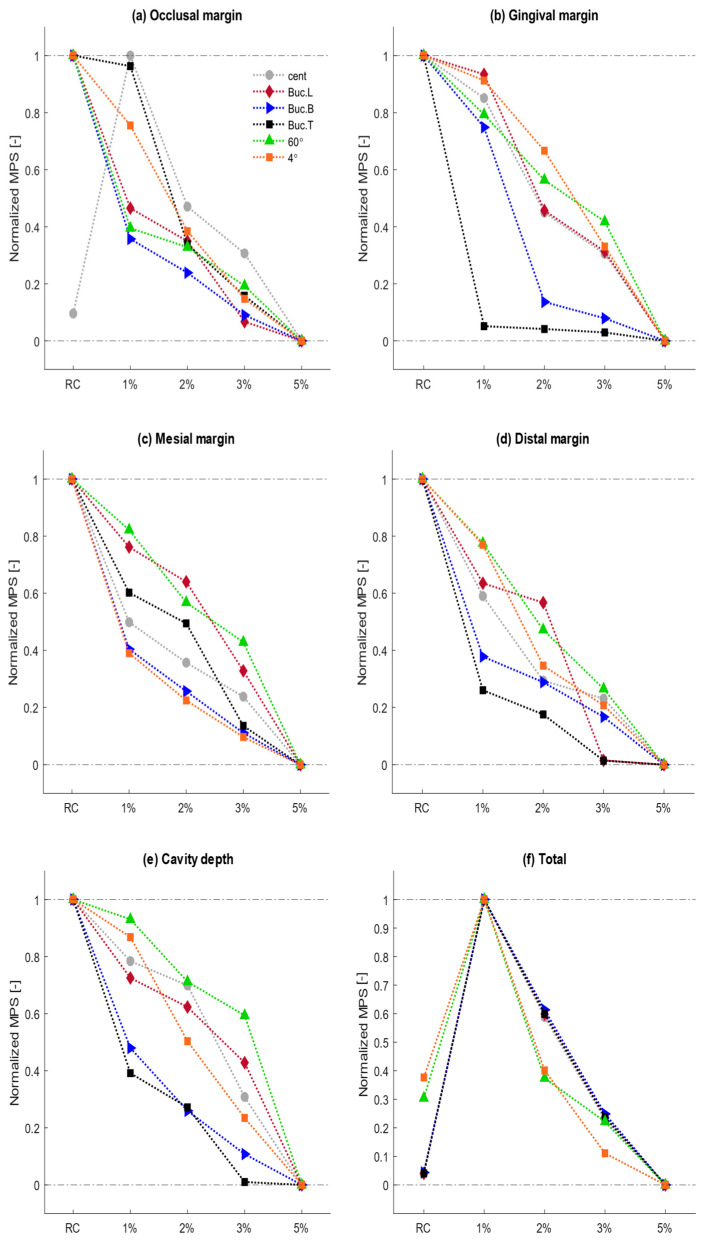
Normalized changing trend of MPS values created among various incorporation ratios of ZnO NPs in various boundary conditions.

Restoring the lesion with RC causes an increase in MPS values at the gingival margin under all boundary conditions. However, other margins show various behaviors under different loading conditions. This is the point where the addition of ZnO NPs is of particular importance. This is due to observing an inverse relation between the content of ZnO NPs and the MPS values at all probe sites. The normalization of MPS values in each boundary condition is used to clearly demonstrate the changing trend while increasing the ZnO NP ratio. Normalized stress curves for each boundary condition with respect to the lowest and highest MPS values are illustrated in Figure 5. Also, the absolute MPS values under various boundary conditions are reported in Table S2. The notable finding is that all MPS values in restoration containing 5% wt. ZnO NPs show the lowest values observed among all restored, and even unrestored cavities.

#### 3.2.2. Thermal Stress Analysis

As the tooth is unrestored, the stresses are more concentrated at lateral margins of the cavity. Restoring the lesion brings about a dramatic increase in MPS values at all probe sites. However, this increase in occlusal and mesial margins is more significant in comparison with other probe sites.

MPS values observed in 3% and 5% groups are lower than the RC group in both thermal conditions. The cold thermal condition results in higher stress accumulation in the restoration part compared with the hot one.

## 4. Discussion

The cervical region of the tooth is prone to damage due to destructive loadings. Thus, evaluating the biomechanics and stress distribution in this area betters the understanding of clinical failures. Knowing about conditions and materials which result in higher stress accumulation helps us minimize the problems encountered in daily practice. In previous studies, the stress distribution in different classes of lesions restored with conventional restorative materials is investigated. However, the behavior of modified materials containing nanoparticles has not been assessed yet. Therefore, here we use 3D FEM to analyze the stress distribution and values in cervical restorations containing ZnO NPs. We assess the performance of these modified composites by changing different varieties to be well-informed about the optimal incorporation ratio.

Distribution of stress on the tooth-restoration complex plays a pivotal role in the durability and success rate of the restoration. High stress values might result in treatment failure and consequent problems for patients. Hence, it is essential to consider these parameters while restoring dental lesions. The MPS, as well as the VMS, are known as the gold standards for evaluating stress distributed in tooth structure. VMS is defined as a scalar quantity obtained from the stresses acting on any structure. It considers all axial planes to measure the overall stress distribution on the tooth-restoration complex, while MPS indicates the stress accumulated in a specific area under uniaxial loading when the basis of other stress tensors is zero [45]. Thus, in this study, the stress distributions in composites containing various ratios of ZnO NPs are analyzed in both VMS and MPS units.

To conduct this study, a tooth with the highest potential risk for incidence of cervical lesions among various types of teeth is chosen. The mandibular first premolar is introduced as the most common tooth for NCCLs due to its special anatomy and transitional position in the jaw [46]. In general, the teeth are exposed to various environmental conditions which produce stresses in them [11]. The stress concentration is usually observed at the cervical region, which causes treatment failure [47]. Aside from accumulated stresses, bacterial agents are another etiology of failures due to increasing secondary caries. ZnO NPs are widely use in dentistry and many studies have been conducted to assess the properties of dental materials containing these NPs. However, their behavior in various thermo-mechanical conditions is still an unanswered question. Hence, we decided to investigate the thermo-mechanical stresses accumulated in restorations containing ZnO NPs. The results demonstrated different stress values in various studied materials.

When it comes to stress analysis, mechanical properties of materials play a pivotal role. The Young’s modulus is a decisive factor in mechanical behavior and, thereby, the retention rate of restorations. Strong correlation between marginal failure and the Young’s modulus of materials is reported in cervical restorations [30]. Our results indicate different patterns of stress accumulation at various probe sites. Material with a higher Young’s modulus (RC + 1wt.% ZnO NPs sample) demonstrates better behavior in lateral margins and depth of the cavity. Some studies also report the advantage of utilizing materials with a higher Young’s modulus [12,30]. Considering MPS analysis, negative stress values (indicating compressive stress) are mostly accumulated at cavity depth and the distal margin. Thus, better performance of materials with a higher Young’s modulus is due to demonstrating higher resistance to deformation and producing lower stress values in adjacent areas. In contrast, material with a lower Young’s modulus (RC + 5 wt.% ZnO NPs sample) enables better stress distribution in occlusal and gingival margins and the restoration part in our study. Regarding positive MPS values indicating tensile stress accumulation at occlusal and gingival margins, materials with a lower Young’s modulus enable better stress distribution. This is the result of having more tendency to flex with the tooth rather than being debonded. This finding is consistent with previous studies [17,42,48,49]. It should be mentioned that no significant difference between the retention rates of various restorative materials is also reported by Brouning et al. [50]. In this clinical trial, despite implicating two materials with widely different Young’s modulus values, no difference between the retention rates is observed after 12 months. However, it is obvious that the accumulation of higher stress increases the incidence of fatigue and failure over time. Finding no difference between retention rate of restorations in this study may be attributed to the time period and loading conditions in which the samples are studied.

It is evident that various factors affect the retention of the restorations. Thus, considering a single component does not accurately reflect the actual behavior of the tooth. However, a greater difference between the restoration and the tooth structure properties causes more stress concentrations and discontinuities. A photo-elastic examination of the stress distribution within the cervical lesions reveals that the stress is mostly concentrated at the apex of the unrestored lesions. Being restored with composites, the concentrated stress at the apex and the cervical lesion in the lingual region is relieved, while the stress values increase at the gingival and occlusal margins [51]. These findings are in line with the results of our study. The stresses caused by occlusal forces tend to develop NCCLs due to stress concentration at the lateral borders and the depth of the unrestored cavity. By restoring them, more resistance is created against tooth flexion. Thus, the progression of the NCCLs is prevented. Being restored, the stress values in the occlusal and gingival margins are increased. This increase is more dramatic at the gingival margin due to its proximity to bone. Also, given that the difference in the Young’s modulus of the restorative material and the dentin is much greater than its difference with the enamel, this increase in stress in the gingival margin is expected. A higher stress value is a potential source of damage to the adhesive layer, resulting in microleakage and debonding. Thus, the gingival margin should be given particular consideration to prevent failures. Regarding the results, the material with a lower Young’s modulus (5 wt.% models) seems to be more suitable for restoring cervical cavities due to less stress concentration at the gingival border and restoration part.

Another etiological factor for abfraction is loading site and magnitude. Loadings applied at the outside of the middle third region of the tooth are known as a cause for crack formation which could result in abfraction [41]. Besides, the farther the loading site is from the fulcrum of the tooth, the more destructive stress is created in tooth structure. Thus, the upper third of the buccal cusp (functional cusp of mandibular premolar) is chosen to properly indicate loadings that create more stress at the cervical region of the tooth. The angle of applied oblique loads is considered 45° to represent parafunctional forces in lateral movements of the jaw [35,40,41]. It has been reported that the normal occlusal forces are around 150 N and hyper-functional forces range from 240 to 290 N [43]. So, the magnitude of 200 N has been considered as a proper force to imitate occlusal forces. The impact of various loading conditions on stress distribution in the cervical region of the tooth is investigated in previous studies [35,42]. Rees reports that the maximum stress at the cervical region is observed under loadings on the internal slope of cusp. Moreover, central forces mimicking CR movements cause a decrease in accumulated stress in the lower third of the tooth [35]. However, assessing the impact of loading conditions on stresses in cervical cavities restored with modified composites had not been investigated previously. Congruent with Rees’s study, we observe that the overall stress in the restoration and gingival margin show the maximum and minimum values under Buc.L and Cent loadings, respectively. So, it is concluded that the most suitable forces are the ones with parallel orientation.

For thermal stress analysis, the initial temperature and cold/hot boundary conditions are designated as 36.5, 4, and 60 °C, uniformly regarding Palmer’s study [44]. In restored teeth, higher thermal expansion coefficients of restoration compared with the tooth structure cause different expansions. Consequently, the restored models illustrate higher stress values compared with unrestored ones. Clinical studies show an increase in structural damage, particularly in the bonding systems and cracks, which causes microleakage and discoloration of the composites under various thermal conditions. Thus, using materials with lower differences in the thermal expansion coefficients is recommended due to generating lower stress values [11,36,52]. In our study, adding ZnO NPs does not make a significant difference in thermal properties of the base material. However, among studied ratios, 3 and 5 wt.% samples indicate better performance due to having lower thermal expansion coefficients, which is consistent with the abovementioned studies.

In addition to thermal stresses, we also assess the temperature changes within the tooth-restoration structure. Similar to Çelik’s study, the temperature at cavity depth remains constant due to the distance from the applied stimuli and low thermal conductivity of resin-based materials [36]. Besides, Çelik reports that the surface temperature within 2 s is similar in tooth structures and different restorations. However, we observe the lowest temperature changes in the 5 wt.% model in both thermal conditions. This difference is attributed to implicating the static state environment in our study. Moreover, Çelik denotes that lower thickness of the enamel causes the maximum stress concentration in the gingival region and makes it prone to cracks after aging. However, we observe lower stress at the gingival border than other margins. This difference could be resulted from considering the gingival margin in dentin, assessing different teeth and materials in our study. In addition, Guler et al. report that the cold exposure (5 °C) causes higher stress than the heat exposure (55 °C) in the restored tooth due to the greater difference between initial and final temperatures [11]. These results are consistent with our findings. However, in contrast with our study, Guler reports that increasing the temperature creates more stress. This difference could be attributed to different study conditions and considering thermal and mechanical stimuli simultaneously in Guler’s study.

Although FEM is the most accurate method for achieving the values and patterns of thermo-mechanical stress distributions, it does not represent the actual behavior of the tooth in vivo. This is owing to the limitations in the reconstruction of biological properties and dynamic forces [32,36]. The success/failure rate of dental treatments is affected by a variety of factors and properties of materials. Considering the antimicrobial properties, L N Niu et al. report that the optimal antibacterial behavior of a restorative material after aging was seen in models containing 5 wt.% ZnO NPs, in comparison with higher percentages (10 wt. % ZnO NPs) [53]. Besides, higher contents of NPs inversely affect the structural integrity of the restoration. Thus, the incorporation ratios up to 5% wt. are chosen to be investigated in this study. We consider some assumptions based on previous studies to reduce the possibility of error and obtain more clear stress distribution patterns in the tooth-restoration complex. First, the effect of pulp and spongy bone were neglected in order to eliminate their stress-absorbing effect [30,35]. Secondly, the average dimension of 0.2 mm for meshes is considered and a mesh convergence test with an error of less than 10% is performed to ensure that the mesh does not affect the results. This dimension of meshes provides the opportunity of discriminating the stress values with slight differences properly [54]. Moreover, we tried to have a correct interpretation and comparison of even small differences in stress values by normalizing the data. Among study and control groups, the 5 wt.% sample caused lower stress values (or no significant difference with RC model in some margins) in all probe sites under both thermal and mechanical conditions. All that said, a reduction in the incidence of secondary caries, the progress of NCCLs, and the debonding of restorations is expected in clinical practice due to showing better antimicrobial and thermo-mechanical properties. However, a comprehensive viewpoint assessing various properties of these materials is of importance to analyze their costs and benefits for clinical application. Further investigations with different study conditions (different ZnO NPs ratios, testing methods, material type, and loading conditions) are required to achieve better material choice.

## 5. Conclusions

In this study, we found out that under all investigated mechanical loadings, the 5 wt.% model presents the best mechanical performance. In hot/cold thermal conditions, the 3 and 5 wt.% models indicate better thermal behavior than pure composite. Applying central forces and lingual loads on the buccal cusp results in minimum and maximum stress values in cervical restoration, respectively. The cold stimulus creates higher thermal stress in the restored models than the hot stimulus. Restoring the cervical lesions results in a decrease in the VMS values in the lateral margins and cavity depth, and an increase in the occlusal and gingival margins. Among studied ratios, an inverse relation is observed between the ZnO NPs content of composite restoration and the MPS values accumulated in the tooth restoration interfaces.

## Figures and Tables

**Table 1:** Thermal and mechanical properties of the structures and materials used for simulating the finite element models.

Materials	Young’s Modulus (GPa)	Poisson’s Ratio	Coefficient of Thermal Expansion $\left(\frac{1}{{}^{\circ}C}\right)\times 10^{-5}$	Specific Heat (J/g °C)	Thermal Conductivity (W/m °C)
Enamel	84.1	0.33	1.70	0.75	9.2
Dentin	18.6	0.31	1.06	1.17	6.3
Bone	13.7	0.3	1.00	1.84	5.8
Periodontal ligament	0.012	0.45	0.4	0.36	5
Resin Composite (RC)	3.2	0.3	3.70	0.82	0.11
RC + 1% ZnO NPs	3.5	0.3	3.67	0.81674	0.1199
RC + 2% ZnO NPs	3.6	0.3	3.63	0.81348	0.1298
RC + 3% ZnO NPs	3.4	0.3	3.60	0.81022	0.1397
RC + 5% ZnO NPs	3.7	0.3	3.53	0.8037	0.1595

## Data Availability

The data presented in this study are available within the article and in Tables S1 and S2.

## Supplementary Materials

The following supporting information can be downloaded at: https://www.mdpi.com/article/10.3390/ma15165504/s1, Table S1: The absolute VMS values under various boundary conditions at six different probe sites; Table S2: The absolute MPS values under various boundary conditions at six different probe sites; Figure S1: Von Mises stress distribution (MPa) under vertical load of 200 N on buccal cusp tip. (A) Buccal view, (B) Mid-cut view; Figure S2: Von Mises stress distribution (MPa) under 2 central vertical loads of 100N. (A) Buccal view, (B) Mid-cut view; Figure S3: Von Mises stress distribution (MPa) under buccally oriented oblique load of 200 N on buccal cusp. (A) Buccal view, (B) Mid-cut view; Figure S4: Von Mises stress distribution (MPa) under 60 °C thermal condition; Figure S5: Von Mises stress distribution (MPa) under 4 °C thermal condition.
