# Supplementary material for: Mechanical and Thermal Stress Analysis of Cervical Resin Composite Restorations Containing Different Ratios of Zinc Oxide Nanoparticles: A 3D Finite Element Study
Source: Materials (Basel). 2022 Aug 10;15(16):5504. doi: 10.3390/ma15165504 (PMC9412397; doi:10.3390/ma15165504)
Supplement: Supplementary file 1 [file materials-15-05504-s001.zip › Table S1.pdf]

VMS values								
	Boundary conditions	material	Occlusal margin	Gingival margin	Mesial margin	Distal margin	Cavity depth	Total
Mechanical stress analysis	Buc.T	cavity	6.6157	2.9754	93.174	95.589	46.089	-
		RC	14.532	53.738	26.005	71.862	8.933	96.714
		1%	16.443	55.02	23.885	64.154	5.426	101.02
		2%	15.355	54.592	24.453	68.07	6.151	99.143
		3%	14.345	54.074	25.36	70.655	7.594	97.574
		5%	13.942	53.653	26.148	72.804	8.9935	96.536
	Buc.B	cavity	10.167	8.8799	57.686	8.3033	19.538	-
		RC	13.555	30.816	46.366	7.3985	12.453	41.126
		1%	16.441	33.049	40.836	4.6881	10.926	41.825
		2%	14.165	32.576	44.19	5.721	11.251	41.544
		3%	13.92	31.166	45.894	6.8558	11.921	41.277
		5%	13.011	30.719	46.69	7.5452	13.09	41.095
	Buc.L	cavity	4.7826	5.1373	182.18	131.98	74.063	-
		RC	11.722	99	82.395	92.449	11.019	203.42
		1%	13.122	100.72	72.682	78.976	7.4048	215.08
		2%	12.641	99.826	77.35	80.749	8.8145	210.21
		3%	12.168	99.225	80.232	87.961	9.8599	205.82
		5%	11.448	98.708	83.698	93.273	11.217	202.92
	Cent	cavity	1.6682	1.094	15.259	15.733	8.4651	-
		RC	2.7914	2.9237	7.99928	11.916	2.7278	17.852
		1%	3.151	3.1637	5.8789	10.857	1.2365	19.43
		2%	2.9478	3.0994	6.4462	11.222	1.3567	18.76
		3%	2.7878	3.0847	7.5461	11.569	1.9094	18.169
		5%	2.7483	2.982	8.1774	12.072	2.511	17.787
Thermal stress analysis	˚60C	cavity	8.8122	0.3988	14.307	14.592	1.3384	-
		RC	38.318	26.387	32.856	54.845	11.197	107.74
		1%	38.34	26.429	32.696	54.151	10.79	126.67
		2%	38.1	26.626	32.376	54.154	9.9411	110.57
		3%	37.93	26.554	32.004	53.162	9.4236	97.787
		5%	37.636	25.605	31.797	52.754	9.2553	85.419
	˚4C	cavity	3.7284	0.18558	15.334	10.578	0.6662	-
		RC	54.74	19.974	62.052	53.071	14.046	150.06
		1%	56.448	18.65	61.569	51.665	14.257	175.14
		2%	55.024	17.783	60.836	51.041	13.979	152.82
		3%	52.004	17.192	59.664	50.384	13.42	135.1
		5%	48.585	16.419	58.276	46.105	12.936	117.67

Table S1. supplementary: The absolute VMS values under various boundary conditions at six different probe sites.
