# Supplementary material for: Mechanical and Thermal Stress Analysis of Cervical Resin Composite Restorations Containing Different Ratios of Zinc Oxide Nanoparticles: A 3D Finite Element Study
Source: Materials (Basel). 2022 Aug 10;15(16):5504. doi: 10.3390/ma15165504 (PMC9412397; doi:10.3390/ma15165504)
Supplement: Supplementary file 1 [file materials-15-05504-s001.zip › Table S2.pdf]

| MPS values | | | | | | | | |
|---|---|---|---|---|---|---|---|---|
| | Boundary conditions | material | Occlusal margin | Gingival margin | Mesial margin | Distal margin | Cavity depth | Total |
| Mechanical stress analysis | Buc.T | cavity | 9.5195 | 2.0314 | 4.2334 | -0.49453 | -0.58881 | - |
| | | RC | 7.5788 | -6.5427 | 1.986 | 0.98168 | 2.6811 | 21.325 |
| | | 1% | 7.5303 | -2.1684 | 1.5479 | 0.42335 | 2.2134 | 23.686 |
| | | 2% | 6.7132 | -2.1224 | 1.4293 | 0.35935 | 2.1213 | 22.7 |
| | | 3% | 6.4769 | -2.067 | 1.034 | 0.23815 | 1.9204 | 21.81 |
| | | 5% | 6.27 | -1.9305 | 0.885 | 0.22697 | 1.9125 | 21.225 |
| | Buc.B | cavity | 10.728 | 9.0365 | 7.398 | 7.7382 | 23.607 | - |
| | | RC | 6.0571 | 30.395 | 28.919 | 6.9574 | 6.8231 | 37.303 |
| | | 1% | 4.6493 | 29.731 | 27.185 | 5.6687 | 5.9561 | 37.83 |
| | | 2% | 4.39 | 28.115 | 26.757 | 5.483 | 5.5891 | 37.617 |
| | | 3% | 4.065 | 27.9635 | 26.3325 | 5.2312 | 5.3366 | 37.416 |
| | | 5% | 3.8669 | 27.7547 | 26.0102 | 4.8856 | 5.1566 | 37.279 |
| | Buc.L | cavity | 3.624 | 2.0219 | 2.717 | 1.8424 | -1.2664 | - |
| | | RC | 6.019 | -2.7603 | 6.3537 | -0.34775 | 3.3919 | 59.812 |
| | | 1% | 4.65 | -2.6659 | 5.4494 | -0.22246 | 3.2678 | 65.762 |
| | | 2% | 4.3538 | -1.983 | 4.9877 | -0.19915 | 3.2219 | 63.274 |
| | | 3% | 3.6319 | -1.7774 | 3.8038 | -0.00975 | 3.1339 | 61.031 |
| | | 5% | 3.46 | -1.3285 | 2.5589 | -0.0045 | 2.9406 | 59.56 |
| | Cent | cavity | 1.5973 | 0.17026 | 0.99476 | 0.3166 | -0.14322 | - |
| | | RC | 3.3944 | -0.46647 | 0.19215 | 0.41447 | 0.69212 | 5.8793 |
| | | 1% | 3.7439 | -0.4368 | 0.15368 | 0.32513 | 0.63708 | 6.5746 |
| | | 2% | 3.5392 | -0.35702 | 0.14281 | 0.26059 | 0.61548 | 6.2764 |
| | | 3% | 3.4758 | -0.32823 | 0.13366 | 0.2469 | 0.51588 | 6.0169 |
| | | 5% | 3.3573 | -0.26756 | 0.11542 | 0.1966 | 0.43737 | 5.8511 |
| Thermal stress analysis | ˚60C | cavity | 0.023365 | 0.071108 | 0.26084 | 0.42052 | 0.052661 | - |
| | | RC | 29.59 | 3.3961 | 27.287 | 10.16 | 8.9412 | 24.365 |
| | | 1% | 23.465 | 2.8571 | 25.013 | 9.0121 | 8.8891 | 33.89 |
| | | 2% | 22.787 | 2.259 | 21.769 | 7.4648 | 8.7259 | 25.322 |
| | | 3% | 21.415 | 1.8787 | 19.99 | 6.4096 | 8.6375 | 23.236 |
| | | 5% | 19.462 | 0.78983 | 14.519 | 5.0562 | 8.1938 | 20.201 |
| | ˚4C | cavity | 0.012996 | 0.04563 | 0.2153 | 0.325 | 0.02363 | - |
| | | RC | 39.661 | 2.223 | 55.391 | 12.325 | 11.0237 | 38.9691 |
| | | 1% | 37.546 | 2.1615 | 50.125 | 11.562 | 10.8696 | 46.2624 |
| | | 2% | 34.344 | 1.9889 | 48.6943 | 10.1596 | 10.4432 | 39.2571 |
| | | 3% | 32.298 | 1.7531 | 47.5891 | 9.6987 | 10.1289 | 35.8615 |
| | | 5% | 31.025 | 1.521 | 46.7569 | 9.0123 | 9.8546 | 34.5648 |

Table S2. supplementary: The absolute MPS values under various boundary conditions at six different probe sites.
