# Supplementary figures and images for: Mechanical and Thermal Stress Analysis of Cervical Resin Composite Restorations Containing Different Ratios of Zinc Oxide Nanoparticles: A 3D Finite Element Study
Source: Materials (Basel). 2022 Aug 10;15(16):5504. doi: 10.3390/ma15165504 (PMC9412397; doi:10.3390/ma15165504)

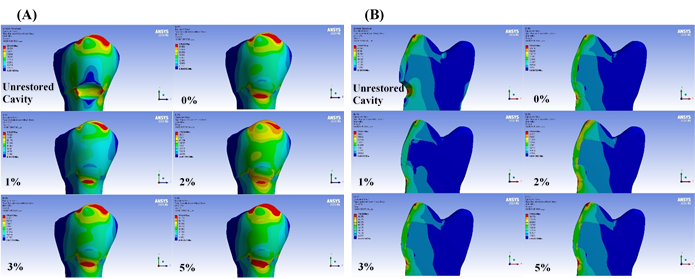

Supplement: Supplementary file 1 [file materials-15-05504-s001.zip › Figure S1.TIF]

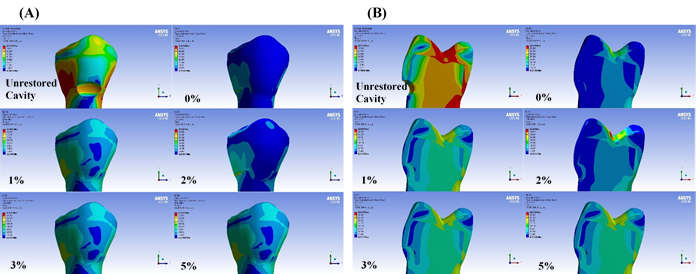

Supplement: Supplementary file 1 [file materials-15-05504-s001.zip › Figure S2.TIF]

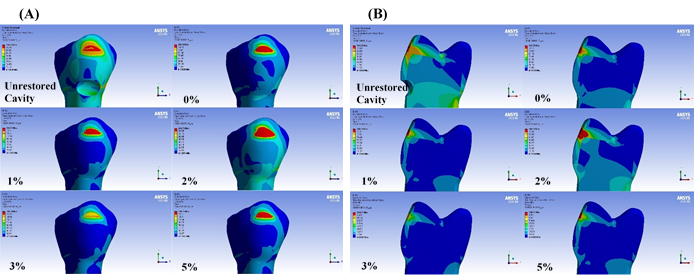

Supplement: Supplementary file 1 [file materials-15-05504-s001.zip › Figure S3.TIF]

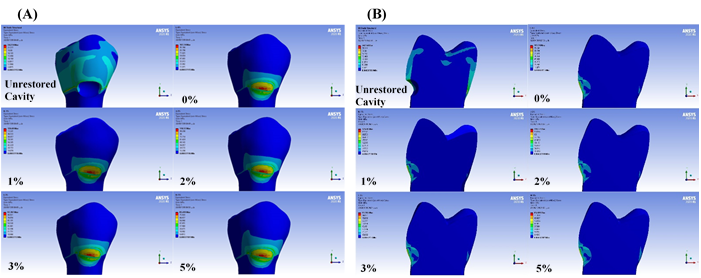

Supplement: Supplementary file 1 [file materials-15-05504-s001.zip › Figure S4.TIF]

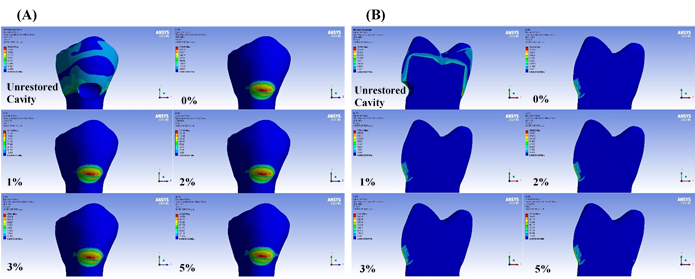

Supplement: Supplementary file 1 [file materials-15-05504-s001.zip › Figure S5.TIF]
